# Neonatal Adaptation in Infants Prenatally Exposed to Antidepressants- Clinical Monitoring Using Neonatal Abstinence Score

**DOI:** 10.1371/journal.pone.0111327

**Published:** 2014-11-03

**Authors:** Lisa Forsberg, Lars Navér, Lars L. Gustafsson, Katarina Wide

**Affiliations:** 1 Department of Pediatrics, Karolinska University Hospital, Stockholm, Sweden; 2 Department of Clinical Science, Intervention and Technology (CLINTEC), Karolinska Institutet, Stockholm, Sweden; 3 Department of Laboratory Medicine, Division of Clinical Pharmacology, Karolinska Institutet, Stockholm, Sweden; Medical University of Vienna, Austria

## Abstract

**Background:**

Intrauterine exposure to antidepressants may lead to neonatal symptoms from the central nervous system, respiratory system and gastrointestinal system. Finnegan score (Neonatal Abstinence Score, NAS) has routinely been used to assess infants exposed to antidepressants in utero.

**Aim:**

The purpose was to study neonatal maladaptation syndrome in infants exposed to selective serotonin reuptake inhibitors (SSRI) or serotonin-norepinephrine reuptake inhibitors (SNRI) in utero.

**Method:**

Retrospective cohort study of women using antidepressants during pregnancy and their infants. Patients were identified from the electronic health record system at Karolinska University Hospital Huddinge containing pre-, peri- and postnatal information. Information was collected on maternal and infant health, social factors and pregnancy. NAS sheets were scrutinized.

**Results:**

220 women with reported 3^rd^ trimester exposure to SSRIs or SNRIs and who gave birth between January 2007 and June 2009 were included. Seventy seven women (35%) used citalopram, 76 used (35%) sertraline, 34 (15%) fluoxetine and 33 (15%) other SSRI/SNRI. Twenty-nine infants (13%) were admitted to the neonatal ward, 19 were born prematurely. NAS was analyzed in 205 patients. Severe abstinence was defined as eight points or higher on at least two occasions (on a scale with maximum 40 points), mild abstinence as 4 points or higher on at least two occasions. Seven infants expressed signs of severe abstinence and 46 (22%) had mild abstinence symptoms. Hypoglycemia (plasma glucose <2.6 mmol/L) was found in 42 infants (19%).

**Conclusion:**

Severe abstinence in infants prenatally exposed to antidepressants was found to be rare (3%) in this study population, a slightly lower prevalence than reported in previous studies. Neonatal hypoglycemia in infants prenatally exposed to antidepressant may however be more common than previously described.

## Background

Psychiatric conditions are common during and after pregnancy. A large US study showed a prevalence of 13% for both mood and for anxiety disorders in pregnant or postpartum women [1]. Antidepressants are commonly used to treat major depressive disorders as well as other psychiatric conditions such as anxiety and obsessive compulsive disorders. Selective serotonin reuptake inhibitors (SSRIs) are the most prescribed group of antidepressants, also in pregnant women [2]. Serotonin norepinephrine reuptake inhibitors (SNRIs) are also used during pregnancy with similar effects on prenatally exposed children as SSRIs [3]. A population based register study showed that 3% of all pregnant women in Sweden used antidepressants, mainly SSRIs, three months prior to conception, whereas the numbers decreased during pregnancy, down to 1% in the third trimester [2]. In Denmark, there has been an increase in antidepressant use in pregnancy. In 1997, 0.2% of all pregnant women had at some point during pregnancy been using antidepressants, in 2010 this figure had increased to 3.2% [4].

Use of paroxetine during early pregnancy has been linked to an increased risk of heart malformations, OR 1.66 (95% CI 1.09 to 2.53) and hypospadias, OR 2.45 (95% CI 1.12 to 4.64) [5]. A Canadian study found a significantly increased risk of heart malformations only in infants exposed to paroxetine daily doses higher than 25 mg [6]. A neonatal maladaptation syndrome in infants exposed to SSRIs during late pregnancy is well known. It includes symptoms such as jitteriness, feeding problems, respiratory distress, hypoglycemia [5], [7], [8]. The relative risk of persistent pulmonary hypertension, a potentially life threatening condition, is increased in infants prenatally exposed to SSRI, from 1.2 per 1000 live births in unexposed infants to 3 per 1000 live births in SSRI exposed neonates [9]. Maternal illness (depression, anxiety) may also contribute to milder neonatal symptoms usually resolving within a week [10]. The mode of action for neonatal maladaptation after SSRI/SNRI exposure is largely unknown. ‘Abstinence’ due to the discontinued distribution of the pharmacological substance at delivery as well as serotonergic overstimulation has been suggested [8].

Finnegan score, or Neonatal Abstinence Score sheet (NAS) was originally developed to diagnose abstinence in infants prenatally exposed to opioids [11], but has also been used to assess neonatal symptoms in SSRI exposed infants [12].

As stated above, antidepressants during pregnancy is a common clinical problem, of growing significance. Conducting clinical research in the field of perinatal pharmacological exposure is complicated due to many ethical considerations and an abundance of potential confounders. This study was initiated to shed light on the occurrence of neonatal abstinence/maladaptation after SSRI/SNRI exposure *in utero* and its prevalence, timing, severity and clinical features of this known but inadequately described condition.

## Methods

The study design was a retrospective cohort study. The study patients were identified in the integrated electronic health record used in the delivery and prenatal care units as well as for outpatient maternal visits (Obstetrix^R^ 2.12.01.100, Siemens AG, Munich, Germany). Patients with diagnostic codes for psychiatric illness during pregnancy and exposure to fetus of pharmacological substances were selected as the study population.

Women fulfilling these criteria and who had been giving birth at the Karolinska University Hospital Huddinge between 1 January, 2007 and 30 June, 2009 were included. Patients with reported substance abuse (alcohol or drugs) or use of certain kinds of neurotropic medication (antiepileptic drugs, lithium, opioids) were excluded. Information about substance abuse was reported in the patient records but not systematically confirmed through urine toxicology or breathalyser. Patients where substantial parts of the prenatal care records could not be retrieved were also excluded.

Information regarding social situation, prenatal medication (antidepressants and other pharmacological substances), health status and pregnancy related complications were retrieved from the prenatal part of the electronic health record (Obstetrix^R^). The information on use of antidepressants, bensodiazepines and other pharmacological substances was based on self-report or stated by the prescribing physician in the patient records. Obstetrix^R^ was also used to extract clinical information about the delivery, infant health including health problems in the maternal ward and admission to the neonatal ward. During the study period, all infants where the mother had reported antidepressant use during late pregnancy were routinely observed at the maternal ward for at least 72 hours and modified Finnegan score used regularly to detect signs of abstinence. The assessments were extracted from the electronic health record system and analyzed.

**Figure 1:**
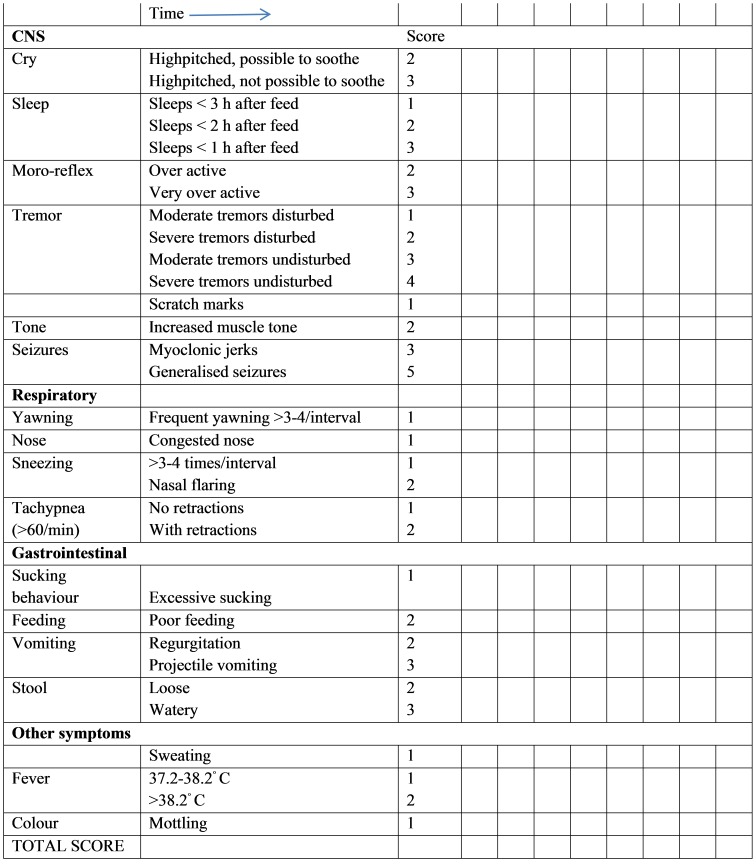
Neonatal Abstinance Score. Modified from Finnegan to Swedish, (Sarman 2000) here re-translated.

A modified Finnegan score or Neonatal Abstinence Score (NAS) in Swedish was used in this study [13]. A version re-translated into English from Swedish is showed in Fig. 1. The assessment includes four categories: central nervous system (CNS), respiratory, gastrointestinal and ‘other symptoms’. The maximum score for each scoring occasion is 40 points, of which 21 are related to CNS items. Neonatal abstinence was in this study classified as either mild (score 4 and above on at least two occasions) or severe (score 8 and above on at least two occasions). A similar classification has been used previously [12].

Population data regarding rates of neonatal admission, rates of smoking in early pregnancy, caesarian section and maternal age with regard to the Stockholm county area and Karolinska University Hospital Huddinge were obtained from the Swedish Neonatal Quality Register (SNQ) and from the statistical unit at National Swedish Board of Health and Welfare. These data were obtained as processed data [14].

Statistical analyses were performed with Statistica^R^ 64, version 12 (StatSoft Scandinavia AB, Uppsala, Sweden). In the comparison between groups, chi square Pearson's test, ANOVA (Analysis of Variance) or Kruskal Wallis was used. Ordinal regression was used in the comparison of different levels of abstinence. Logistic regression or linear regression was used in the multiple regression models.

The study was a retrospective evaluation, based on patient records and conducted several years after the delivery of the infants. Neither written nor verbal informed consent was obtained. Data from patient records were extracted by one researcher and only relevant parts of the records were entered. The data was recorded in a data file with the confidentiality that is applied to patient records. Every individual was given a code number to strengthen the protection of confidentiality. No individual is possible to identify from the report. The study design was approved by the Regional Ethical Review Board in Stockholm (no. 2010/686-31).

## Results

During the study period of 2.5 years between January 1, 2007 and June 30, 2009, 365 patients with diagnostic code for pharmacological exposure to fetus and psychiatric illness during pregnancy delivered at Karolinska University Hospital Huddinge. Use of SSRI/SNRI was confirmed in 277 patients. Thirty-four were excluded due to confirmed (or strongly suspected) substance abuse, opioid use or use of other psychotropic medication such as lithium or antipsychotics. Twenty patients could not be included due to missing data. One pair of twins was excluded. Two hundred and twenty patients remained; 54 gave birth in 2007, 103 in 2008 and 63 during the first six months of 2009. During 2007, 2008 and 2009 a total of 13755 infants were born at Karolinska University Hospital Huddinge [14].

Table 1 describes the women in the cohort with regard to social factors, health status and reported medication during pregnancy (antidepressants and other); divided by reported third trimester antidepressant use. The most common exposures were citalopram, sertraline and fluoxetine (Table 1). The remaining patients, in all 33 (15%), were exposed to escitalopram (13 patients), venlafaxine (11 patients), paroxetine (8 patients) and duloxetine (one patient). Depression and anxiety disorders were the most common reasons for antidepressant use.

**Table 1:** Characteristics of study population, women with antidepressant treatment and delivery at Karolinska University Hospital Huddinge Jan 2007 to June 2009.

	All antidepressants	Citalopram	Sertraline	Fluoxetine	Other antidepressants**
	n = 220	n = 77 (35%)	n = 76 (35%)	n = 34 (15%)	n = 33 (15%)
***Age of mother at delivery*** mean (years)	31.2	31.2	32.1	29.4	31.0
***Maternal psychiatric diagnosis*** ≠ (n)					
Depression	168	59	58	29	22
Anxiety disorder	80	29	27	8	16
Other psychiatric diagnoses≠≠	34	7	12	10	5
***Tobacco in early pregnancy*** * (n [%])					
No smoking	189 (86%)	69 (90%)	62 (82%)	29 (85%)	29 (88%)
<10 cigarettes per day	25 (11%)	7 (9%)	11 (14%)	4 (12%)	3 (9%)
>10 cigarettes per day	4 (2%)	1 (1%)	2 (3%)	1 (3%)	
Snuff	2 (1%)		1 (1%)		1 (3%)
***Employment status*** * (n [%])					
Full time employed	87 (40%)	32 (42%)	33 (43%)	9 (26%)	13 (39%)
Part time employed	54 (25%)	19 (25%)	18 (24%)	5 (15%)	12 (36%)
Unemployed	72 (33%)	23 (30%)	23 (30%)	18 (53%)	8 (24%)
Missing data	7 (3%)	3 (4%)	2 (3%)	2 (6%)	
***Body mass index*** * mean (kg/m^2^)	24.4	24.0	24.7	24.9	24.1
***Other illnesses*** * (n [%])	106 (48%)	33 (43%)	42 (55%)	12 (35%)	19 (58%)
***Antidepressant dose in third trimester*** *** median (range) (mg)	NA	20 (5–80)	50 (5–200)	30 (10–80)	NA
***Use of benzodiazepine during pregnancy*** (n [%])	14 (6%)	4 (5%)	5 (7%)	3 (9%)	2 (6%)
***Use of other medications during pregnancy*** (n [%])	136 (62%)	43 (56%)	44 (58%)	27 (79%)	22 (67%)
***Mode of delivery*** (n [%])					
Vaginal delivery	155 (70%)	57 (74%)	48 (63%)	25 (74%)	25 (76%)
Cesarean section (planned)	24 (11%)	7 (9%)	11 (14%)	2 (6%)	4 (12%)
Cesarian section (acute)	20 (9%)	5 (6%)	9 (12%)	4 (12%)	2 (6%)
Vacuum extraction	21 (10%)	8 (10%)	8 (11%)	3 (9%)	2 (6%)

≠ one patient can have more than one diagnosis.

≠≠ other psychiatric diagnoses, all antidepressants (n): Phobia (4), Post-traumatic stress disorder (4), Eating disorder (9), Personality disorder (6), Obsessive compulsive disorder (7), Bipolar disorder type II (1), Attention deficit hyperactivity disorder (3).

*stated by mother at interview with midwife in prenatal care center, gestational week 10–14.

**other antidepressants (n = 33): escitalopram (13 patients), venlafaxine (11), paroxetine (8) and duloxetine (1).

***Highest dose in maternal care records during third trimester.

NA  =  not applicable.

Cigarette smoking in early pregnancy in the cohort was 13% (for all 2.5 years) as compared to 5.4% in 2007, 4.9% in 2008 and 4.7% in 2009 for women in early pregnancy in the whole Stockholm area. BMI was higher in the cohort, 24.4 kg/m^2^, compared to all women who gave birth in the Stockholm area 2007 (23.9), 2008 (23.9) and 2009 (23.8).

A total of 48% of the women had ‘other illnesses’ documented in the prenatal care records. Most of these illnesses were mild and manageable, such as musculoskeletal problems, asthma, migraine, thyroid diseases or gastrointestinal reflux disease. Some were potentially serious such as obesity or chronic hepatitis. Eleven patients had more severe conditions such as diabetes mellitus, inflammatory bowel disease, serious or potentially serious pulmonary, rheumatic or neurological diseases.

Birth weight and head circumference did not differ between the four different exposure groups (Table 2). Linear regression adjusting for maternal age, maternal smoking in early pregnancy, gestational age and infant sex showed no statistical significance regarding birth weight (p = 0.75). A model adjusting for only maternal smoking did not reach statistical significance. For head circumference the corresponding result was p = 0.56. A model adjusting for maternal smoking only did not show any statistically significant differences between the four antidepressant exposure groups. Prematurity (gestational age of 36 weeks and 6 days or less) was seen in 13% of the infants exposed to citalopram (Table 2). In a logistic regression model adjusting for maternal age, maternal tobacco use in early pregnancy and infant sex there were no statistically significant differences regarding prematurity between citalopram exposed infants (13%) and infants exposed to sertraline (7%, p = 0.85), fluoxetine (9%, p = 0.67) or other antidepressants (3%, p = 0.30).

**Table 2:** Neonatal characteristics of infants exposed to antidepressants in utero, born 1 Jan 2007 to 30 June 2009 at Karolinska University Hospital.

	All antidepressants	Citalopram	Sertraline	Fluoxetine	Other antidepressants**
	n = 220	n = 77	n = 76	n = 34	n = 33
**Sex (male:female)** (n[%])	106∶114 [48∶52]	38∶39 [49∶51]	36∶40 [47∶53]	16∶18 [47∶53]	16∶17 [48∶52]
**Gestational age** (weeks)					
mean [95% CI]	38.5 [38.3 to 38.7]	38.2 [37.8 to 38.5]	38.8 [38.4 to 39.1]	38.6 [38.1 to 39.1]	38.5 [38.0 to 39.1]
range	31–42	31–41	32–41	35–42	34–42
**Prematurity** (n [%]) *	19 [9]	10 [13]	5 [7]	3 [9]	1 [3]
**Birth weight** (gram)					
mean [95% CI]	3386 [3314 to 3458]	3302 [3177 to 3425]	3476 [3352 to 3600]	3385 [3217 to 3553]	3377 [3167 to 3588]
range	1790–5015	1790–4315	1905–5015	2110–4670	2305–4615
**Head circumference** (cm)					
n	217	75	75	34	33
mean [95% CI]	34.3 [34.1 to 34.5]	34.0 [33.6 to 34.4]	34.4 [34.1 to 34.8]	34.3 [33.7 to 34.8]	34.5 [33.9 to 35.1]
range	28–38	28–37.5	31–38	30–37	31–37.5
**Hypoglycemia** (n [%])	42 [19] †	8 [11]	15 [20]	12 [35]	7 [21]
**Respiratory diagnosis** (n [%])	14 [6]	5 [6]	5 [7]	2 [6]	2 [6]
**Jaundice** (n [%])	10 [5]	5 [6]	3 [4]	1 [3]	1 [3]
**Neonatal care** (n [%])	29 [13]	10 [13]	9 [12]	6 [18]	4 [12]

*gestational age <37+0.

**other antidepressants (n = 33): escitalopram (13 patients), venlafaxine (11), paroxetine (8) and duloxetine (1).

† p 0.02, statistical test (logistic regression, adjusting for gestational age, maternal tobacco use, infant sex and 5 min Apgar).

Hypoglycemia in the neonate was defined as a blood glucose level <2.6 mmol/L. Hypoglycemia was diagnosed in 42 (19%) of all study infants. A logistic regression model (adjusting for 5 min Apgar, gestational age, tobacco use in early pregnancy and infant sex) showed significantly more infants with at least one hypoglycemic episode in the fluoxetine group, 12 (35%), compared to 8 (10%) in the citalopram group, p = 0.01. There were no significant differences between citalopram exposed infants and infants exposed to sertraline (p = 0.74) or other antidepressants (p = 0.70). A model adjusting for only 5 min Apgar and gestational age did not add further information.

The blood glucose values of all hypoglycemic infants (n = 42) ranged from 1.0 to 2.5 mmol/L (mean 2.0 mmol/L). In hypoglycemic infants exposed to citalopram (n = 8) the mean blood glucose was 2.0 (range 1.4 to 2.3). These numbers for hypoglycemic infants exposed to sertraline, fluoxetine and other antidepressants were 1.9 (range 1.0 to 2.5), 1.9 (range 1.0 to 2.5) and 2.1 (range 1.1 to 2.5) respectively. There were no significant differences between the groups, p = 0.6 (Kruskal Wallis).

Respiratory disorders included ‘respiratory distress syndrome of newborn’, ‘transient tachypnea of newborn’, ‘other respiratory distress of newborn’, pneumothorax, persistent fetal circulation and ‘condition originating in the perinatal period’, the latter an apneoic event. There were 14 cases (6.3%) of respiratory disorders and 4 of them were in prematurely born infants. No significant correlation was seen between occurrence of respiratory disorders and type of antidepressant exposure in third trimester, in logistic regression adjusting for 5 min Apgar score and gestational age (in weeks), p = 1.0.

There were five children with Apgar score <7 at five minutes of age.

Jaundice was diagnosed in 10 children (4 born prematurely). There were no significant differences between groups for antidepressant exposure, in a logistic regression model adjusting for 5 min Apgar and gestational age, p = 1.0.

One hundred and fifty-eight infants (71%), received the diagnostic code Z001A ‘healthy infant born in hospital’.

Twenty-nine (13%) of the infants exposed to antidepressants were admitted to the neonatal ward. During 2007, 2008 and 2009, 13755 infants were born at Karolinska University Hospital Huddinge [14] and 1418 (10.3%) of them admitted to the neonatal ward (data from Swedish Neonatal Quality Register). A univariate analysis showed no statistical difference between exposed and unexposed infants (born at Karolinska University Huddinge) regarding probability for neonatal admission (p = 0.16). Multivariate analysis (logistic regression adjusting for 5 min Apgar and gestational age) did not reveal any differences in admittance rates between the four exposure groups (p = 0.67).

No specific treatment for neonatal maladaptation syndrome was performed. The patients were treated with supportive care. Respiratory disorders were treated with CPAP (continuous positive airway pressure) if needed (8 cases) and hypoglycemia with intensified oral feeding, in some instances with i.v glucose, depending on the severity of the condition.

A total of 205 infants (93%) were scored with Neonatal Abstinence Score sheet (table 3). The mean time at ‘peak score’ i e highest recorded score in patients who had a score of four or higher at any time, was for infants exposed to citalopram 31.6 hours (range 2–90; SD 21.4), for sertraline exposure 37.4 (1–84; 21.7), fluoxetine exposure 37.1 (4–74; 21.3) and other antidepressants exposure 22.1 hours (3–64; 17.6). There were no statistically significant differences between the groups (Kruskal Wallis, p = 0.07).

**Table 3:** Analyses of Neonatal Abstinence Score in infants exposed to antidepressants in utero, born at Karolinska University Hospital Huddinge 1 Jan 2007 to 30 June 2009.

	All antidepressants	citalopram	sertraline	fluoxetine	Other antidepressants≠	p
***Scoring performed*** (n[%])	205 [93%]	71 [92%]	71 [93%]	33 [97%]	30 [91%]	0.5†
***Number of scoring occasions***						
(mean [SD] [range])	8.4 [3.0] [2–27]	8.5 [2.7] [3]–[18]	8.4 [2.9] [2]–[20]	8.4 [4.4] [2–27]	8.3 [2.2] [5]–[13]	0.66††
***Neonatal abstinence*** (n[%])						
*No abstinence*	152 [74%]	51 [72%]	53 [75%]	24 [73%]	24 [80%]	
*Mild abstinence* *	46 [22%]	18 [25%]	16 [23%]	7 [21%]	5 [17%]	
*Severe abstinence* **	7 [3%]	2 [3%]	2 [3%]	2 [6%]	1 [3%]	0.85***

*two or more scorings of 4–7.

**two or more scorings of 8 or above.

***Multiple ordinal regression, adjusted for infant sex and 5 min Apgar.

† Chi square test.

†† Kruskal Wallis.

≠ other antidepressants (n = 33): escitalopram (13 patients), venlafaxine (11), paroxetine (8) and duloxetine (1).

CNS symptoms contributed with on average 67% of the points in the group with severe abstinence (two or more scores of eight or higher) and 58% in the group with mild abstinence (two or more scores of four or higher). Infants exposed to citalopram had mild abstinence in 18 cases (25%), severe abstinence in two cases. Comparing citalopram to sertraline exposure in ordinal regression, adjusting for 5 min Apgar and infant sex, infants exposed to sertraline had an odds ratio of 1.08 (95% confidence interval, 0.64 to 1.82). Odds ratio for fluoxetine compared to citalopram was 0.86 (95% CI, 0.45 to 1.63) and for other antidepressants compared to citalopram 1.3 (95% CI, 0.61 to 2.61). There were no significant differences in the occurrence of mild or severe abstinence between the different types of antidepressant exposure, adjusting for 5 min Apgar and infant sex, p = 0.85.

There were no significant differences in mean maternal SSRI dose in third trimester (analysis made for citalopram [p = 0.20], sertraline [p = 0.83] and fluoxetine [p = 0.18]) between infants with or without abstinence (Mann Whitney U test).

Twenty-nine infants in the study were admitted to the neonatal ward. Twenty four percent of them were not scored with NAS compared to 4% of the infants who remained in the maternity ward (p = 0.0007).

## Discussion

This study shows that a majority of all infants born to mothers with SSRI or SNRI treatment during pregnancy are healthy in the neonatal period. Only 3% developed a severe abstinence syndrome and 22% signs of mild abstinence, the symptoms mainly arising from the central nervous system. Our results are in accordance with other studies with similar or slightly higher prevalence of the neonatal abstinence/maladaptation syndrome in infants with intrauterine SSRI/SNRI exposure. A study that used Finnegan score and similar definitions of severe and mild neonatal maladaptation syndrome found mild or severe neonatal maladaptation syndrome in 18/60 (30%) infants exposed to SSRI, compared to 0/60 control infants [12]. A slightly different definition of severe maladaptation (NAS score >12 or 3 scores >8) reported a prevalence of 4% in infants exposed to SSRI and 9% in infants exposed to venlafaxine [15]. Oberlander et al described symptoms of transient poor neonatal adaptation in 30% of a group of infants exposed to SSRI with or without the addition of clonazepam [16]. The reasons behind the differences in prevalence may be found in the different definitions of neonatal maladaptation syndrome as well as differences in study population (maternal age, socioeconomic status, health care, concomitant pharmacological treatment, use of tobacco and alcohol).

Hypoglycemia was common, 19% of all infants had blood glucose of 2.6 mmol/L or lower. Most of them were treated with intensified oral feeding. Other studies have found an association between prenatal antidepressant exposure and hypoglycemia [7]. The etiology behind this may be increased demands of energy in babies with abstinence. The prevalence reported in other studies have been lower, 1.4% in a register based cohort study [17] or 3/60 exposed infants in an Israeli cohort [12]. These large differences between studies may be explained by different definitions of hypoglycemia (the ICD-10 diagnosis of neonatal hypoglycemia, P70.4, requires blood glucose 2.2 mmol/L or lower) as well as differences in study protocols or in clinical practice. Hypoglycemia is also fairly common in all infants. In a clinical study of healthy term and preterm infants, 12% of the term and 14% of the preterm infants had a blood glucose of below 2.6 mmol/L during the first week of life [18]. In observational studies such as this one, the high rates of hypoglycemia may also be due to the fact that jitteriness and tremor can be perceived as symptoms of hypoglycemia leading to the measurement of blood glucose, increasing the chance of diagnosing a low blood glucose value. Hypoglycemia in infants is however a potentially serious condition [19], [20] and needs to be further investigated, preferably in a randomized controlled study. Until then, screening of blood glucose in neonates prenatally exposed to antidepressants should be considered.

The mothers in this study had a more unfavorable health status than the average pregnant Stockholm County population with regard to higher proportion of smokers and higher BMI. This may also affect neonatal outcome. The catchment area of Karolinska University Hospital Huddinge includes many socioeconomically constrained areas, often associated with higher BMI and a higher proportion of smokers. Any comparison between this catchment area and the rest of Stockholm County must be done with caution. Studies on infants to mothers with opioid use during pregnancy have shown increased risk of neonatal abstinence as well as other negative neonatal outcomes in opioid exposed infants where there was a concomitant heavy cigarette consumption [21]. Smoking cessation and control of weight gain during pregnancy may be an important method in this patient group to improve infant (and maternal) health.

Several other studies report a high incidence of respiratory symptoms in infants exposed to SSRIs [5], [22]. However, some experts argue that the increased risk of neonatal complications that have been attributed to SSRI exposure may be due to confounding factors [23]. Fourteen neonates in this study had a respiratory diagnosis but the study design did not allow a comparison to an unexposed population.

Different antidepressants have different pharmacokinetic properties. This may, in theory, influence the timing of symptoms of abstinence or serotonergic overstimulation in exposed neonates. Time to peak value (NAS) was therefore analyzed but failed to show any significant differences between the groups. Individual factors such as metabolic and transporter capacity of SSRI/SNRI in mother and child as well as placenta may be of greater influence on the occurrence of maladaptation and timing of symptoms [15], [24]–[26]. Time to peak value ranged between 2 and 90 hours suggesting that infants exposed to antidepressants may have a relatively late onset of peak abstinence symptoms. With today's extremely short duration of hospital stay for newborns, the symptoms may even occur after discharge from hospital. Karolinska University Hospital Huddinge, as well as several other hospitals, has abandoned the policy of advising parents of infants prenatally exposed to antidepressants to stay at least 72 hours. In uneventful deliveries, the mother and infant usually leave the maternity ward within 48 hours.

Seven percent of the infants in the study were not subject to abstinence scoring. This may be due to the fact that the midwife nurse was not aware of the guidelines, that the infant was perceived to be healthy or that the scoring sheet was not properly stored. Also, NAS is often used as a screening tool and may therefore be considered unnecessary in sick infants in the neonatal ward who are subjected to more sophisticated surveillance methods. This may explain why scoring was not performed in a larger proportion of the infants admitted to the neonatal ward compared to the maternity care units.

The causal relationship between SSRI or SNRI exposure and high scoring values in the NAS is of course not established in each infant included in this study. Other illnesses or prenatal risk factors, unrelated to pharmacological exposure or maternal illness, may contribute. This study could not investigate an increased risk of neonatal symptoms in infants prenatally exposed to antidepressants compared to unexposed ones.

It can be argued that we recruited a too small study population. There may have been differences between the exposure groups had they been larger. The retrospective design of the study and the changed policy of the hospital (Neonatal Abstinence Score and increased surveillance of all infants prenatally exposed to SSRIs were abandoned in July 2009) made it impossible to perform this study on a larger cohort. The retrospective design is of course an important limitation to this study. A prospective design would have allowed us to for example confirm antidepressant use and exposure in mother and infant.

Monitoring neonates with prenatal exposure to drugs and abuse substances is a delicate task. There is a risk that health care professionals are being too interventional, introducing unnecessary concern regarding the infant's health. There is also a risk, with antidepressants becoming increasingly common in the pregnant population- and considered ‘safe’- that knowledge about potentially serious complications is lost. A modified Neonatal Abstinence Score could be useful in detecting abstinence/maladaptation in this group. The CNS and respiratory categories are probably more useful than the gastrointestinal category. Most important is however knowledge among care givers regarding the possible symptoms that can arise after prenatal exposure to antidepressants.

## Conclusions

Severe abstinence in a cohort of infants exposed to SSRI/SNRI in late pregnancy was rare since it occurred in only 3% of the cases. Neonatal hypoglycemia was observed in 19% of the infants which is higher than previously reported. Admission to the neonatal ward was seen in 13% of the infants and did not differ significantly from the admittance rate in other infants born in the same hospital during the studied period.
